# On the Medical Qualities of the Asclepias Syriaca

**Published:** 1852-06

**Authors:** George W. Allen

**Affiliations:** Mississippi


### WESTERN JOURNAL

OF

### MEDICINE AND SURGERY.

JUNE, 1852.

Art. I.—*On the Medical Qualities of the Asclepias Syriaca:* an Inaugural Dissertation, submitted to the Trustees and Medical Faculty of the University of Louisville. By George W. Allen, M. D., of Mississippi.

My attention was called to this medicine by my preceptor, Dr. A. E. Thomas, who has made use of the remedy to a considerable extent.

*Description.*—" The Silk Weed has simple stems, from three to five feet high, with opposite lanceolate oblong, petiolate leaves, downy on their under surface. The flowers are large, of a pale purple color, sweet scented, and arranged in nodding umbels, which are two or three in number. The nectary is bidentate. The pod or follicle is covered with sharp prickles, and contains a large quantity of silky seed down, which has been sometimes used as a substitute for fur in the manufacture of hats, and for feathers in beds and pillows.

This species of asclepias is very common in the United States, growing in sandy fields, on the road sides, and on the banks of streams from New England to Virginia. It flowers in July and August. It gives out a white juice when wounded, and hence has received the name of "milk weed," by which it is frequently called." Such is the description of this plant given by Dr. Wood.

It is also known as the "Bitter Root." The root is the part employed which should be dug up in the latter part of September, after the stalk has become dry, or in the spring before the stalk has shot up. As found in the shops it is of various sizes, from that of a small quill to that of the little finger. In length it is from two to six inches, cylindrical, more or less wrinkled, longitudinal and brittle. It is composed of a thick exterior cortical portion covered with a thin epidermis, and presenting, when freshly broken, a lighter color than that of the exposed surface. Its medicinal virtues are communicated to boiling water and to alcohol.

Silk weed is a cathartic, emetic, tonic, and alterative. Possessing, as I think it does, the property of calming irritation and diminishing nervous excitability, it appears to be especially applicable in diseases connected with derangement of the hepatic system, and of the digestive organs generally.

It is held by some to be a fine emmenagogue. I myself made use of the remedy in two cases of difficult menstruation, one of fifteen months' and the other of two years' standiug, with entire relief to both patients.

Within the last eight months I have seen it prescribed by Dr. Thomas in a number of uncomplicated cases of intermittent fever, given at night in sufficient quantity to insure its action on the bowels, and the following day in small doses at intervals; and never in a single instance did it disappoint his expectations.

I have also witnessed the happiest results from its use in the treatment of chronic rheumatism.

It may be given in substance, or in the form of extract, infusion, or tincture. The dose of the powdered root is from ten to thirty grains; of the tincture, prepared by macerating two ounces of the pulverized root in one pint of diluted alcohol, from a half to three drachms; of the extract, from three to ten grains.

I am persuaded, though unable to specify the cases in which its employment is specially indicated, that it may often be advantageously substituted for, or combined with iodine in the treatment of scrofula.

I have had opportunities of closely observing the beneficial results from its administration in three or four cases of this affection. I will mention but one:

Orry, a slave, aged about twenty-two years, and of delicate constitution. Her father is a mulatto of dissipated habits, and has been laboring under a syphilitic taint for many years. The mother is a slight but healthy woman. The patient has ever been delicate and fragile, evidently showing the strumous diathesis. In the fall of 1849, she was under the treatment of Dr. C——, for hepatitis. His treatment was a mercurial course, combined with blisters, tonics, etc. After being under treatment some months she was discharged, with the symptoms much ameliorated, but with the caution that the disease might return. During the following summer and autumnal months she enjoyed a tolerable state of health, but on the return of cold and wet weather, she was taken with the symptoms of scrofula, enlargement of the glands of the neck, the lips and conjunctiva became swollen to their utmost tension; so much so as to become inverted. The whole system became disordered, and the utmost emaciation ensued.

Dr. Thomas's treatment, at first, was that most in vogue for the cure of this affection, viz: iodine, iodide of potassium, arsenic, etc., combined with cod-liver oil.

The most active and prominent symptoms were for a time allayed, but soon resumed their activity; and her general health was such as to induce her owner to believe that she was incurable.

Dr. Thomas having met with success in the treatment of a similar case by the silk weed, determined to try it in this instance. Two grains of the extract were given night and morning, gradually increasing the dose to five grains. This, in conjunction with a proper nourishing diet, brought about, in the course of two weeks, a decided improvement in the condition of the patient. This simple treatment was continued with but little variation for about four months, by which she was gradually restored to health, and she is at this time enjoying better health than she has done for five years, weighs more than she ever did before, and is able to do her full task.

I have also witnessed the happiest results from the use of asclepias in dyspepsia. In one instance, a gentleman who had been dyspeptic for several years, and, to use his own language, "had swallowed a small drug store," was prevailed upon to try the silk weed, and, contrary to his own expectations, he was in a short space of time much benefited. He became fond of the remedy and substituted it for his tobacco, and in the end was relieved of all dyspeptic symptoms. The following is a case which I deem worthy of notice:

Mr. B., a farmer, aged 38, accustomed to regular exercise, and to indulge in the good things of the table; of sanguine temperament and strumous habit. About the beginning of 1845, according to his own account, he began to complain of indigestion, characterized by loss of appetite and strength, by profuse perspiration on making slight muscular exertion, and by increasing emaciation. He was treated actively, and received by different hands a variety of prescriptions, but with only one effect—the aggravation of his sufferings. After long trial and many disappointments, he left his home for the various watering places, but found no relief. In the fall of 1848, he went to Cuba in the hope of regaining his health. He was however disappointed, and after a trial of five mouths of the climate and practitioners, he returned to his home, depressed in mind and wasted in body. On his arrival, in April, 1849, he was pale and emaciated, his countenance and manner indicated both mental and bodily suffering; his appetite was depraved and he was disposed to indulge it; he was suffering from headache, flatulence, and palpitations; from constant restlessness of mind, and was particularly suspicious of the presence of consumption. His skin was pale and sallow, tongue loaded, bowels irregular, and the evacuations unhealthy. He was incapable of mental application, and had well nigh abandoned all hope of being restored to health. Such was the condition of the patient when he applied to Dr. Thomas for relief. After learning the full particulars of the case Dr. T. determined to put him upon the silk weed. He accordingly commenced with half a drachm of the tincture twice a day, and gradually increased the dcse to two drachms. His diet was regulated, which consisted of milk, farinaceous vegetables, and animal food in small quantity. In four weeks from the commencement of this mode of treatment, the general appearance of the patient was much improved, and the dyspeptic and nervous symptoms were less distressing.

A perseverance in the same mode of treatment was strictly enjoined, and the patient continued gradually to improve in looks and spirits. He regained his lost powers; his manner and conduct no longer indicated that he thought only of himself and his ailments; and in the course of a few months, under the silk week, he regained his lost flesh and was restored to health.

February, 1852.

